# A facile preparation of superhydrophobic L-CNC-coated meshes for oil–water separation

**DOI:** 10.1039/d1ra02291a

**Published:** 2021-04-13

**Authors:** Jingda Huang, Mengmeng Li, Youwei Lu, Changying Ren, Siqun Wang, Qiang Wu, Qian Li, Wenbiao Zhang, Xianmiao Liu

**Affiliations:** School of Engineering, Zhejiang A&F University Hangzhou 311300 China zwb@zafu.edu.cn; Center for Renewable Carbon, University of Tennessee Knoxville Tennessee 37996 USA swang@utk.edu; International Center for Bamboo and Rattan Beijing 100102 China

## Abstract

A superhydrophobic stainless steel mesh (called “mesh” in short) is an ideal device to solve oil pollution accidents by oil–water separation. However, its widespread application is prevented by complicated preparation, weak durability, and particularly poor mechanical strength. It is well known that the used adhesives play a key role in the mechanical strength of superhydrophobic coatings. In this study, polyvinylidene fluoride (PVDF) and polydimethylsiloxanes (PDMSs) were respectively used as adhesives and lignin-nanocellulose crystal (L-CNC) particles as main structure materials to prepare L-CNC coated superhydrophobic meshes. Moreover, the meshes coated with L-CNC/PVDF and L-CNC/PDMS were compared with respect to the properties of wettability, sandpaper abrasion, oil–water separation, *etc.* The results showed that the L-CNC/PVDF-coated mesh had a higher water contact angle (WCA = 154.2°) than the L-CNC/PDMS-coated one (WCA = 152.6°), but worse abrasion resistance. Both of them showed high-efficiency oil/water separation with collection rates above 94.5% and stable reusable ability as the oil collection rates for toluene was still above 93.8% after reusing thirty times, meanwhile showing good heat, UV, acid and alkaline resistance properties.

## Abstract 2

A superhydrophobic stainless steel mesh (called “mesh” in short) is an ideal device to solve oil pollution accidents by oil–water separation.

## Graphical abstract

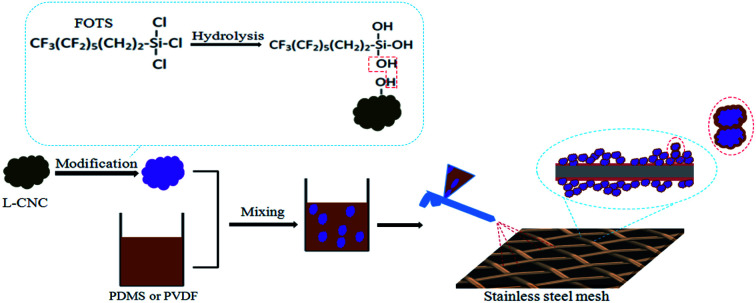

## Introduction

Superhydrophobic surfaces originally inspired by lotus leaves^1^ are well-known for their excellent functions such as waterproof, anti-fouling, corrosion-resistance, self-cleaning, anti-drag, and oil–water separation^2^ and have been widely reported in recent years. It is indispensable for superhydrophobic surfaces to meet both the requirements of reasonable rough structures (usually micro-nano layered structure) and low surface free energy.^3^ Superhydrophobic surfaces could be fabricated on solid substrates such as wood,^4^ metal,^5,6^ textile,^7,8^ ceramics,^9^ and paper^10^*via* numerous methods such as chemical vapor deposition,^11^ sol–gel technique,^12^ etching,^13^ layer-by-layer assembly,^14^ and spraying.^15^ The superhydrophobic stainless steel mesh (called “mesh” for short) is an ideal device to solve increasingly severe oil pollution accidents by oil–water separation^16^ due to its low cost and high convenience. Etching is a common method used to enhance roughness to prepare superhydrophobic meshes.^17^ However, it needs high-cost equipment or a multi-step operation.^18^ Spraying is one of the easiest-to-use ways to prepare superhydrophobic surfaces because of no limitation to materials of any kind or shape. In addition, after hydrophobic modification, inorganic nanoparticles such as SiO_2_, TiO_2_, and CaCO_3_ are often used as main components of spray paint.^19^ Moreover, natural organic materials show a growing trend in utilization and development in numerous fields and are advocated all over the world due to their incomparable environmental performance and rich source.^20^ Nanocellulose crystals (CNCs) are an ideal natural organic material due to their excellent strength, high crystallinity, and large specific surface area.^21^ Lignin-coated cellulose nanocrystal (L-CNC) particles, a commercial product containing lignin of ∼3–6 wt%, have appropriate size, rough surface, and high intensity and show natural advantages in the preparation of superhydrophobic surfaces.^22^

Although superhydrophobic surfaces have numerous advantages and convenient preparation, they suffer from high cost, weak durability, and poor mechanical abrasion-resistance, thus limiting their extensive application.^23^ Especially, the poor mechanical abrasion-resistance is identified as the hardest and most urgent question to deal with.^24^ In addition to improving the mechanical abrasion-resistance of superhydrophobic surfaces, it is indispensable to enhance their adhesion and improve the stability of their surface rough structure.^25^ To achieve this goal, adhesives are absolutely crucial. There is a lot of research done to improve the mechanical properties of superhydrophobic surfaces with adhesives. Such as epoxy resin was used to fix hydrophobic carbon nanotubes to the substrates to form a stable superhydrophobic surface, which showed excellent abrasion-resistance.^26^ Also, hydroxyl acrylic resin could play a stabilizing role between hydrophobic modified SiO_2_ and substrates.^27^ Besides, PVA,^28,29^ PDMS,^30,31^ double-sided tape,^32^ and PVDF^33^ were involved in the enhancements of superhydrophobic surfaces.

Different adhesives have different properties and could provide adhesion with different strength, which would make the mechanical stability of the superhydrophobic surfaces distinct. In our previous study, the effects of epoxy resin and double-sided tape on the abrasion resistance of superhydrophobic coatings were compared. The results showed that both the epoxy resin and double-sided tape had different influence on the stability of L-CNC-based coatings and their microstructure exhibits different damage state after sandpaper abrasion.^22^ However, the preparation needs a multi-step operation, where the substrate is first treated with adhesive, followed by fixing hydrophobic L-CNC particles to the adhesive surface by rolling. In addition, the method is inconvenient for irregular surfaces and the binding force was lacking among L-CNC particles, resulting in the easy collapsing of the surface rough structure.^22^

Therefore, it is very necessary to construct a system where the adhesion between particles and substrates as well as the binding force among particles exist simultaneously and to have a comparation on the stability of the systems with different adhesives. And different adhesives have different enhancement effects on such systems and are rarely taken for comparison, which is significant to select correct adhesives. In this study, to have a guide for the selection of adhesives used in superhydrophobic surfaces, the superhydrophobic L-CNC-coated meshes were constructed only by a one-step spray. In addition, polyvinylidene fluoride (PVDF) and polydimethylsiloxanes (PDMS), two different highly hydrophobic and adherent polymers, were used to build the system as described above, respectively, followed by comparing their abrasion-resistance and oil–water separation abilities.

## Experimental

### Materials

Lignin-coated cellulose nanocrystal (L-CNC) particles, brown powder and lignin content of ∼3–6 wt% were purchased from American Process Inc., Atlanta, GA, USA. Polydimethylsiloxanes (PDMS, (C_2_H_6_OSi)_*n*_, Sylgard 184 silicone elastomer) including its curing agent, was purchased from Dow Corning Inc. Polyvinylidene fluoride (PVDF, –(C_2_H_2_F_2_)_*n*_–, average *M*_w_ = ∼534 000) and 1*H*,1*H*,2*H*,2*H*-perfluorooctyltrichlorosilane (FOTS, CF_3_(CF_2_)_5_(CH_2_)_2_SiCl_3_, 97%) were purchased from Sigma, China. *N*,*N*-Dimethylformamide (DMF, >99.8%, AR) was purchased from Alfa Aesar. Stainless steel meshes with a pore diameter of 175 μm were used as substrates and washed with fresh ethyl alcohol before use.

### Hydrophobic modification of L-CNC particles

As described in our previous study,^22^ L-CNC particles (2 g) and toluene (50 mL) were poured into a 200 mL beaker and stirred using a magnetic stirrer (500 rad min^−1^) for 10 min. Then, FOTS (1 g) was added to the above mixture, followed by continuous stirring for more than 4 h under sealed conditions. Subsequently, the modified L-CNC particles were washed with fresh DMF to remove unreacted FOTS and by-product produced during the modification process. Finally, the modified L-CNC particles were obtained by centrifuging and drying in an oven with 100 °C for 2 h.

### Preparation of the L-CNC/PDMS (or PVDF) hydrophobic mixture

The modified L-CNC particles and PDMS (containing the agent and *m*_PDMS_ : *m*_curing agent_ = 10 : 1) were added to DMF by the mass ratio of 10 : 1 and stirred for 2 h to get the L-CNC/PDMS hydrophobic DMF solution, where the L-CNC/PDMS mixture took up 10% of the total mass. A certain amount of PVDF powder was added to DMF and stirred at 40 °C (500 rad min^−1^) for 3 h until the powder was completely melted to get a PVDF DMF solution of 10 wt%. Further, the L-CNC/PVDF hydrophobic mixture (*m*_PVDF_ : *m*_L-CNC_ = 1 : 10) was prepared by the same method as that used to prepare the L-CNC/PDMS hydrophobic mixture.

### Preparation of superhydrophobic L-CNC/PDMS (or PVDF)-coated meshes

To prevent L-CNC particles from depositing, the L-CNC/PDMS (or PVDF) mixture was stirred for 5 min before use and then sprayed onto the washed stainless steel meshes by an airbrush (Uxcell mini 0.5 K3 HVLP, aotl tools Guangzhou Co., LTD., China) with a nozzle diameter of 0.5 mm. The used pressure (at the range of 10–50 MPa) was supplied by a pint-sized air compressor and the spray quantity of each spray surface (3 cm × 3 cm) was ∼0.5 g. To get the coatings with consistent thickness as far as possible, the duration of each spray is about 5 s and the distance from the airbrush nozzle to the substrates was maintained about 30 cm. Finally, the coated meshes were dried at room temperature for 30 min, followed by moving into an oven with 110 °C and drying for 2 h to get L-CNC/PDMS-coated meshes (or calcining at 240 °C for 2 h to obtain L-CNC/PVDF-coated meshes).

### Characterization

Surface morphologies of the uncoated and coated meshes were observed *via* scanning electron microscopy (SEM, Zeiss Auriga SEM/FIB crossbeam workstation, Germany). L-CNC particles before and after hydrophobic modification were analyzed *via* Fourier transform infrared spectroscopy (FTIR) with a resolution of 4 cm^−1^ at the scanning range of 400–4000 cm^−1^ by scanning 32 times. Next, the samples for FTIR were prepared by adding L-CNC particles to KBr powder at a ratio of 1 : 100 and pressing into a sheet. Water contact angles (WCAs) and slide angles (SAs) of the uncoated and coated meshes were measured using water droplets of 5–8 μL by a contact angle meter (Shanghai Zhongchen JC2000D, China). The WCA was calculated by the average measurement value at five random/different points. The abrasion-resistance test for the superhydrophobic meshes was as described in our previous study,^21^ and the coated-meshes were placed against a sandpaper and weighed loading of 50 g, followed by moving 20 cm for back and forth movement along the sandpaper each time.

## Results and discussion

### Formation mechanism of superhydrophobic L-CNC/PDMS (or PVDF)-coated meshes

**Fig. 1:**
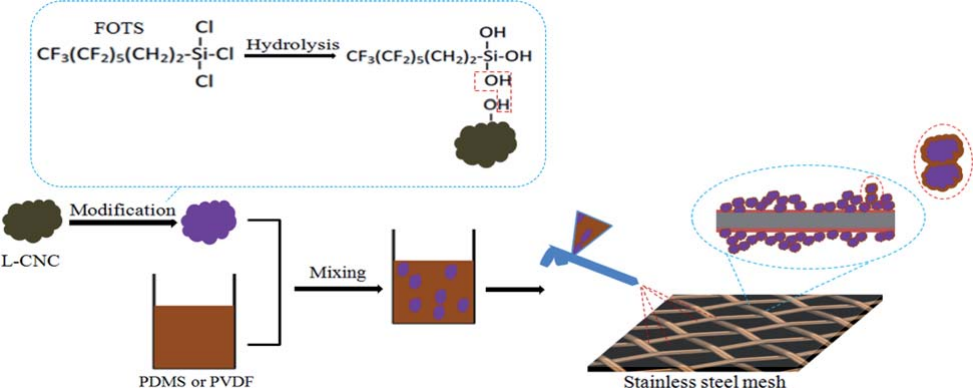
Preparation schematic of the superhydrophobic L-CNC/PDMS (or PVDF)-coated meshes.

Due to the poor surface rough structure, stainless steel meshes are usually treated by chemical etching to give them a suitable rough structure for superhydrophobic surfaces.^34^ Different from etching to enhance roughness, L-CNC particles themselves are uneven and show an irregular rough surface structure, which is just needed for superhydrophobicity.^22^ It is well-known that the untreated L-CNC particles are hydrophilic due to their abundant hydroxyl groups. Therefore, hydrophobic modification is needed. As shown in Fig. 1, first, FOTS as a hydrophobic modifier was hydrolyzed to form FOTS-OH, and then FOTS-OHs were condensed by dehydration to form an FOTS chain. Moreover, a part of hydroxyl groups on the FOTS chain was connected with the hydroxyl groups on the surface of L-CNC particles by dehydration. Finally, FOTS was successfully grafted onto L-CNC particles surfaces. PDMS and PVDF as adhesives were mixed with the modified L-CNC particles, respectively, followed by spraying to get superhydrophobic L-CNC/PDMS (or PVDF)-coated meshes, where L-CNC particles offer the appropriate rough construction, FOTS affords low surface free energy, and PDMS (or PVDF) supplies adhesion and maybe parts of low surface free energy. The reason for choosing both PDMS and PVDF as adhesives is that they are hydrophobic and suitable for the preparation of hydrophobic mixtures, which is conducive to simplify preparation procedures, such as one-step spraying could be used. In addition, not only could PDMS and PVDF provide adhesion between L-CNC particles and substrates but also binding force among L-CNC particles.

### Surface morphologies

**Fig. 2:**
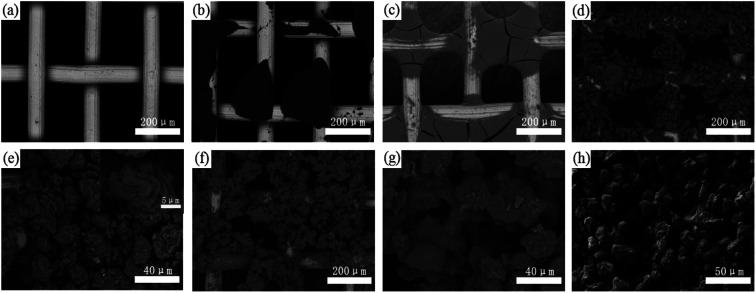
Surface morphologies of the (a) stainless steel mesh, (b) PVDF-coated mesh, (c) PDMS-coated mesh; (d) L-CNC/PVDF-coated mesh and (e) its high magnification; (f) L-CNC/PDMS-coated mesh and (g) its high magnification; (h) the L-CNC coating on the double-sided adhesive.^22^

As shown in Fig. 2a, the untreated stainless steel wires show a relatively clear and smooth surface. After spraying only PDMS (or PVDF) and drying, the wires were not completely covered, but PDMS (or PVDF) contracted into an agglomeration on their surfaces, and PDMS appeared to be cracked (Fig. 2b and c). This is because PDMS (or PVDF) tends to shrink due to the severe shrinkage stress that occurred during high-temperature drying. When treated with L-CNC/PDMS (or PVDF) mixtures, as shown in Fig. 2d–g, the L-CNC particles gathered together to form a “stone flower” like structure on the wires by bonding with PDMS (or PVDF). Moreover, the “stone flower” in the L-CNC/PDMS coated-mesh was larger than that in the L-CNC/PVDF coated-mesh, which might be related to the different properties of PDMS and PVDF and also proved the presence of binding force among L-CNC particles. However, whole wires have not been absolutely covered due to their drying shrinkage, as described above. The high magnification in Fig. 2e shows that the modified L-CNC particles have irregular bumps. In addition, both microscale L-CNC particles themselves and their nanoscale textures on their surfaces formed a micro-nano layered structure required by superhydrophobic surfaces. In our previous study,^22^ L-CNC/double-sided tape (or epoxy) coatings were prepared by rolling the modified L-CNC onto the substrate surface, but the L-CNC particles only piled up instead of forming a “stone flower” like structure (as shown in Fig. 2h) due to the leakage of binding force among L-CNC particles. Therefore, a system where both the adhesion between L-CNC particles and substrates and the binding force among L-CNC particles exist was successfully built.

### Surface wettability

**Fig. 3:**
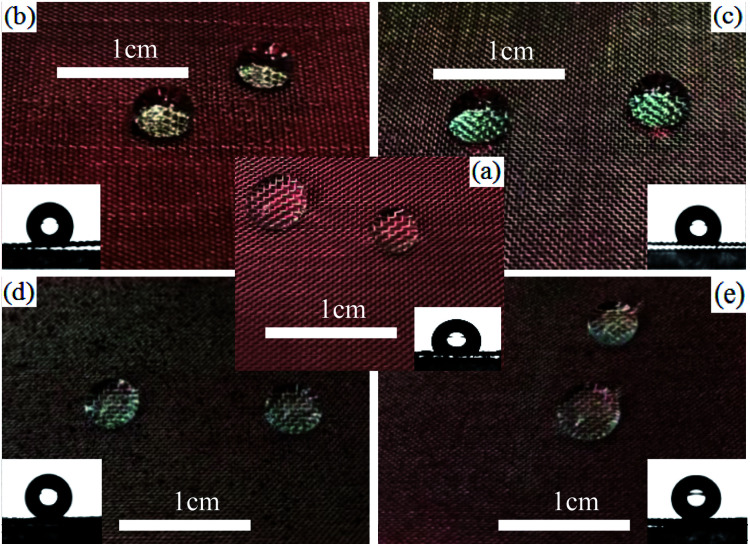
The (a) uncoated mesh, (b) PVDF-coated mesh, (c) PDMS-coated mesh; (d) L-CNC/PVDF-coated mesh, and (e) L-CNC/PDMS-coated mesh.

Surface wettability shows an important influence on the application of solid materials. To better observe the status of water droplets, a piece of red paper was placed under the tested stainless steel meshes. It is well-known that the surface of the untreated mesh is intrinsically hydrophobic^33^ and its WCA is 115.6° (as shown in Fig. 3a). The PVDF (or PDMS)-coated ones show better hydrophobicity and their WCAs on the coated meshes were up to 132.3° and 128.7°, respectively (as shown in Fig. 3b and c). This is because PVDF (or PDMS) itself is a hydrophobic substance and could provide low surface free energy, but fails to realize superhydrophobicity possibly due to the lack of an appropriate rough structure, which is one of the necessary conditions for being superhydrophobic. In addition, the WCA of the PDVF-coated mesh was higher than that of the PDMS-coated one, resulting from the lower surface free energy of PVDF due to the presence of the fluorine element recognized as the element with the lowest surface free energy. After treatment with the L-CNC/PVDF (or PDMS) mixture, the WCAs of L-CNC/PVDF (or PDMS)-coated meshes increased up to 154.2° and 152.6° (as shown in Fig. 3d and e) with SAs of 6° and 8°, respectively, achieving superhydrophobicity due to the appropriate micro-nano layered structure of L-CNC particles and low surface free energy of FOTS.

### FTIR analysis

**Fig. 4:**
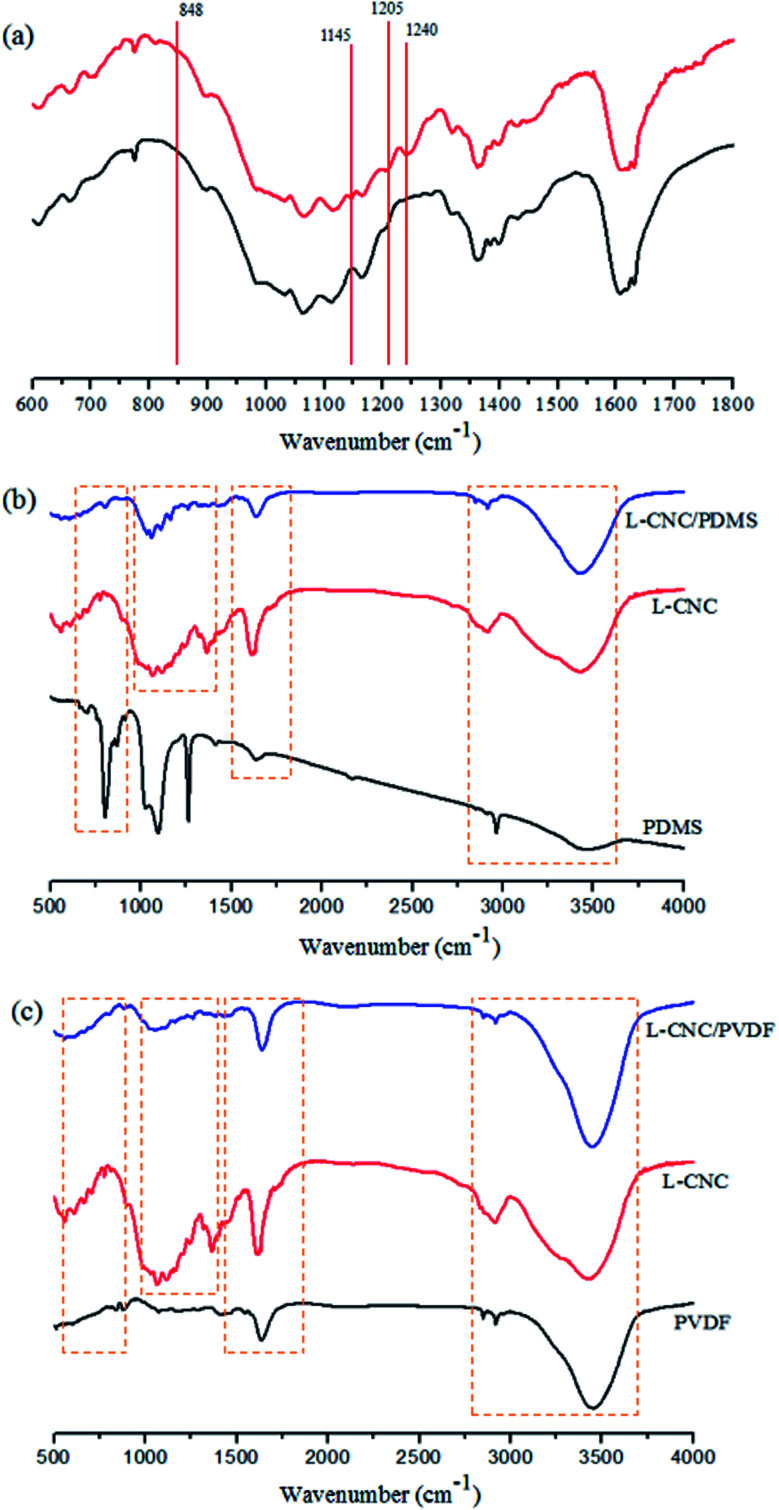
FTIR spectra of (a) the L-CNC before and after modification; (b) PDMS, L-CNC, and L-CNC/PDMS; (c) PVDF, L-CNC, and L-CNC/PVDF.

To verify the change in the chemical composition of the L-CNC particles before and after modification, FTIR test was conducted (as shown in Fig. 4a). The new absorption peaks of the modified L-CNC at 1145 cm^−1^, 1205 cm^−1^, and 1240 cm^−1^ were due to the stretching vibrations of C–F bonds, which came from FOTS and low surface free energy. The new absorption peak at 848 cm^−1^ was formed due to the stretching vibrations of Si–O bonds, which were mainly produced by the dehydration reaction between the hydroxyl group of L-CNC particle surfaces and FOTS-OH, proving that FOTS was successfully grafted onto the L-CNC surface. To further test whether PVDF or PDMS reacts with L-CNC in the process, the contrastive analysis of PDMS (PVDF), L-CNC, and L-CNC/PDMS (PVDF) was conducted. As shown in Fig. 4b and c, compared with L-CNC and PDMS (PVDF), no new absorption peaks are generated in the L-CNC/PDMS (PVDF) mixture, which proves that there is no chemical reaction between L-CNC/PDMS (PVDF), and only physical bonding is present.

### Sandpaper abrasion test

**Fig. 5:**
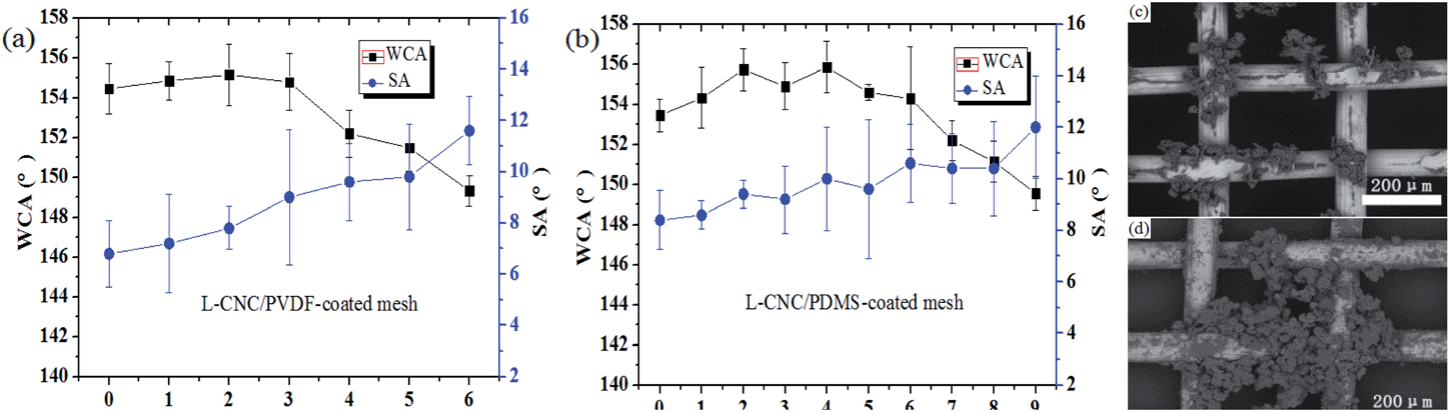
Change in the WCAs of (a) the L-CNC/PVDF- and (b) L-CNC/PDMS-coated meshes with sandpaper abrasion cycles; surface morphologies of (c) the L-CNC/PVDF- and (d) L-CNC/PDMS-coated meshes after end of sandpaper abrasion.

The sandpaper abrasion test is one of the most direct and common ways to characterize the mechanical strength of superhydrophobic surfaces. To facilitate testing, the coated mesh was fixed onto a glass slide with an adhesive tape to make it as flat as possible. The test method was similar to that described in our earlier study.^21^ Briefly, the coated mesh was placed on a sheet of sandpaper (800-grit), which was cut into the same width as the sample and moved 10 cm along the sandpaper under fifty gram loading, followed by moving another 10 cm to go back to the beginning. This is taken as one abrasion cycle. Before the superhydrophobic failure (WCA < 150°), the L-CNC/PVDF (PDMS)-coated meshes could fend against five (or eight) sandpaper abrasion cycles (abrasion length of about 100 cm (or 160 cm)). The results are shown in Fig. 5a and b. It is clear that the L-CNC/PVDF (or PDMS)-coated meshes exhibit a certain ability of abrasion resistance. This mainly depends on the adhesion provided by PVDF (or PDMS) between L-CNC particles and substrate and among L-CNC particles. Normally, the increase in adhesives can enhance its adhesion and then improve abrasion resistance. However, in our early attempts, if the amount of adhesive continues to increase, most of the L-CNC particles are easily covered, leading to superhydrophobic failure. The WCAs continuously decrease as abrasion progresses due to an incessant damage to the surface structure until the removal of some L-CNC particles, resulting in superhydrophobic failure (as shown in Fig. 5c and d). The L-CNC/PDMS-coated mesh exhibits a little better abrasion resistance than the L-CNC/PVDF-coated one, which is mainly related to the adhesion and the properties of the adhesives. Because PDMS after curing is a relatively soft material, but PVDF after curing show a hard surface. While the soft material could buffer parts of external impact force because the soft material could easily occur a shape change and reduce the damage when subjected to an external force, but the hard one not. Therefore, the soft adhesive surface is more beneficial to the abrasion performance.^22^

### Heat resistance test

**Fig. 6:**
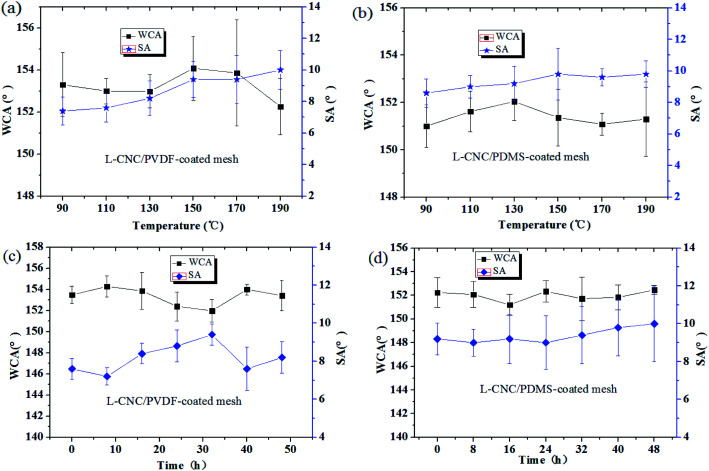
Change in the WCAs of the (a) L-CNC/PVDF- and (b) L-CNC/PDMS-coated meshes exposed to (a and b) different temperature and (c and d) duration of UV radiation.

As described in our previous study,^22^ the heat resistance test was conducted, where the coated meshes were allowed to stay at 90 °C, 110 °C, 130 °C, 150 °C, 170 °C, and 190 °C for 2 h, respectively. As shown in Fig. 6a and b, even up to 190 °C, both of the coated meshes have no significant change in WCAs and remain superhydrophobic, showing good heat resistance. This resulted from two reasons: one is that both PDMS and PVDF are thermosetting resins, which do not melt at the set temperatures and maintain good adhesion; the other reason is that L-CNC particles are of good thermal stability in the temperature range without thermal decomposition, which is attributed to the high pyrolysis temperature of CNC. Because the rough structure constructed by PVDF/PDMS and L-CNC particles is relatively stable, and the superhydrophobicity is not seriously affected.

### UV resistance test

**Fig. 7:**
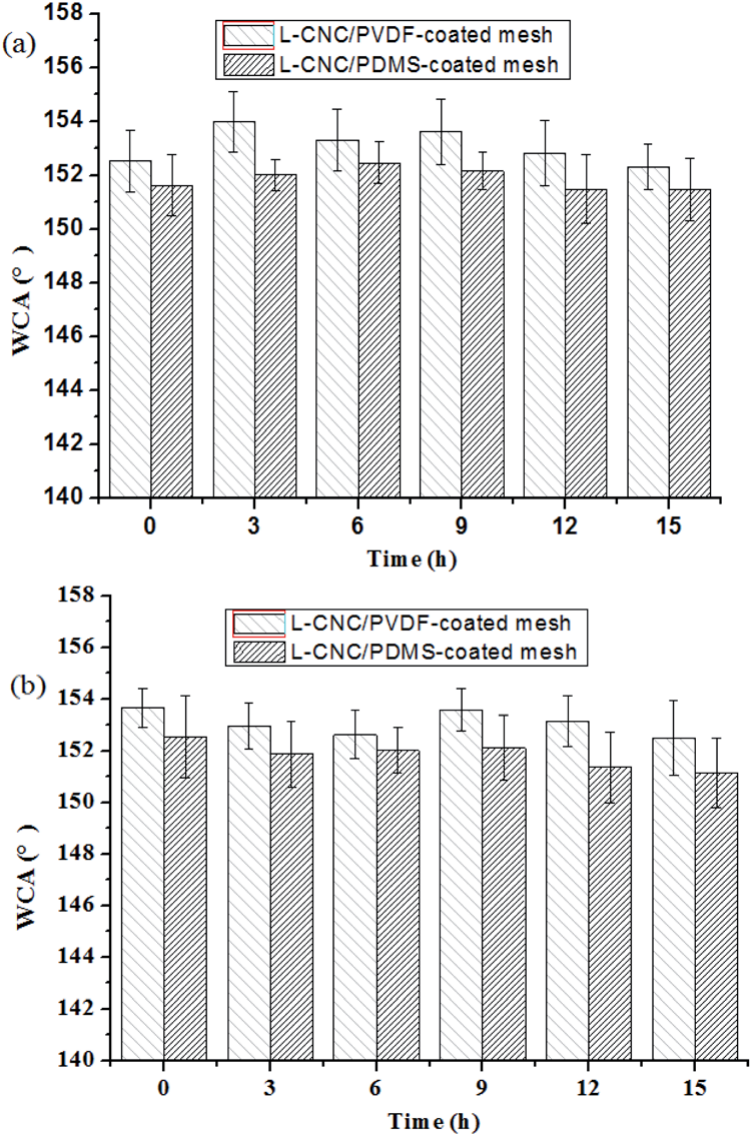
Change in WCAs of the coated meshes in (a) HCl (0.5 wt%) and (b) NaOH (0.05 wt%) solutions.

As described in our previous study,^21^ the coated meshes are exposed to UV radiation (power: 15 W; wavelength: 254 nm) with a distance of 10 cm from the lamp source, and their WCAs are measured every 8 h. It is known that high-strength or extended UV radiation could age materials easily. However, as shown in Fig. 7a and b, the WCAs of both the coated meshes are of irregular change and the difference in the WCAs before and after UV test is tiny and only a maximum change of 2°. This is because CNC is stabile and lignin has excellent UV resistance; furthermore, the C–F bonds in the L-CNC particles have been not damaged by UV radiation.^22^

### Acid and alkaline resistance test

Acid and alkaline resistance is an important index of durability for superhydrophobic surfaces. As described in our previous study,^21^ HCl (0.5 wt%, pH = 1) and NaOH (0.05 wt%, pH = 14) solutions are used to immerse the coated meshes for acid and alkaline resistance tests, and the coated meshes are dried and measured for WCAs every 3 h. As shown in Fig. 7a and b, both of the coated meshes exhibit excellent stability either in acid or an alkaline solution for 15 h and the WCAs remain largely unchanged. This is because L-CNC particles is of high degree of crystallinity and difficult to melt by the acid and alkaline used, and both the cured PVDF and PDMS exhibited good stability and could retain their surface structures without being dilapidated, thus protecting the meshes from corrosion.

### Oil–water separation test

**Fig. 8:**
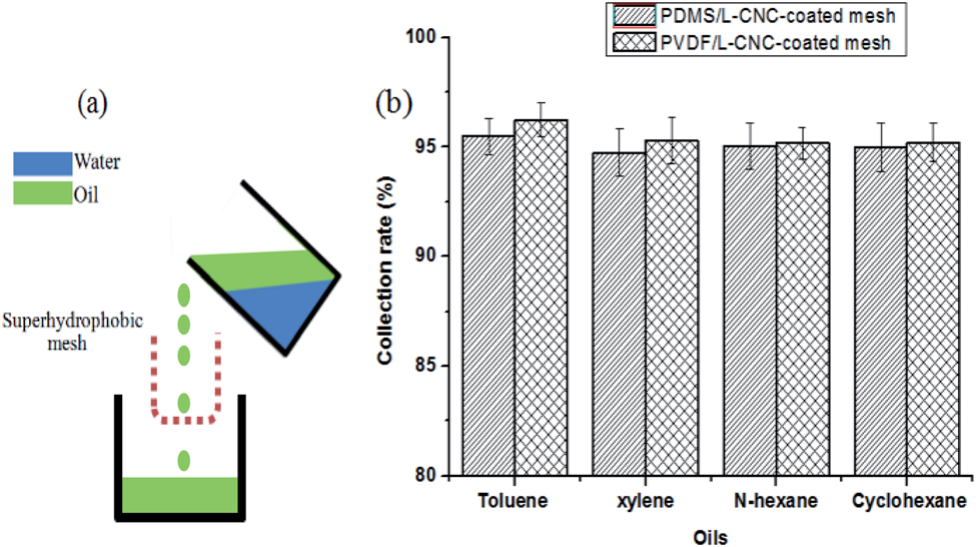
(a) The separation mechanism for the oil/water mixture; (b) the separation efficiency of the L-CNC/PDMS (PVDF)-coated meshes for the separation of the different oil–water mixtures.

As reported in our previous study,^35^ the separation illustration of a coated mesh for the oil/water mixtures is shown in Fig. 8a. When the oil/water mixtures were poured into the “U” shape container bent from the coated mesh, the oil could pass through it into the oil collecting vessel by gravity-induced effect, but the water is intercepted in the “U” shape mesh, resulting in oil/water separation. The collection rate is calculated by the mass of the collected oil to the mass of the oil before the separation. To estimate the separation efficiency of the coated meshes for different oil–water mixtures, four types of oil–water mixtures, such as (toluene, xylene, *n*-hexane, and cyclohexane)–water mixtures, where the volume ratio of oil to water is about equal to 1/1 were used to test. Compared to that obtained in other study^36^ where the separation efficiency of the superhydrophobic mesh is above 94%, L-CNC/PDMS- and L-CNC/PVDF-coated meshes show similar results. As shown in Fig. 8b, the separation efficiency of the L-CNC/PDMS-coated mesh for the four types of oil–water mixtures is above 94.5%, and toluene shows the highest value at 95.49%. In addition, the separation efficiency of the L-CNC/PVDF-coated one reached above 95%, and the highest value also occurred in the toluene and was up to 96.24%. This proves that the coated meshes have excellent separation efficiency.

**Fig. 9:**
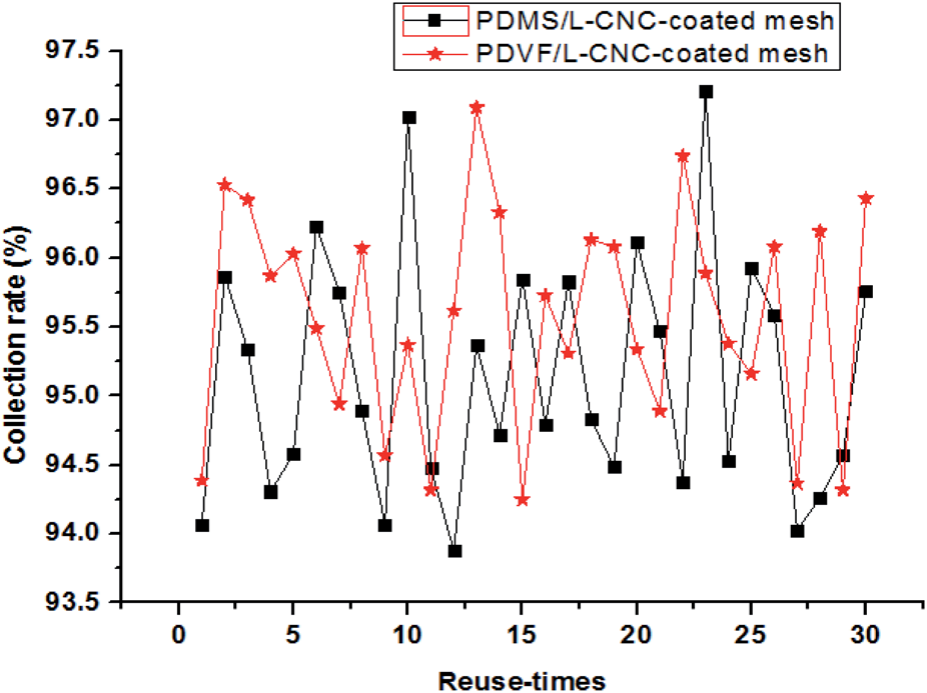
Separation efficiency of the L-CNC/PDMS- and L-CNC/PVDF-coated meshes after reusing thirty times.

To have an assessment on the reusability of the coated meshes, a toluene–water mixture was used to conduct the recycle test. Before each reuse, the coated mesh was cleaned with anhydrous ethanol to remove residual oil and then dried under 60 °C. The results are shown in Fig. 9. The separation efficiency of the L-CNC/PVDF-coated mesh for each time remained above 94.3% and that of the L-CNC/PDMS-coated one remained above 93.8%, proving that the coated meshes possess stable and high-efficiency oil–water separation ability. After reusing for thirty times, the L-CNC/PVDF (or PDMS) coated-meshes were rinsed with absolute ethanol and deionized water to remove residual oil and then dried at 80 °C for 3 h, still maintaining superhydrophobic. This suggests that L-CNC/PVDF (or PDMS) coated-meshes have good recycling performance owing to their stable surface structure, which is because both PVDF and PDMS have a stable adhesion after curing and could resist dissolving in toluene.

## Conclusions

The superhydrophobic L-CNC-coated meshes could be successfully prepared by one-step spraying technique using PDMS and PVDF as adhesives, respectively. PDMS (or PVDF) could provide the adhesion between L-CNC particles and substrates as well as among L-CNC particles in the coated meshes, where a “stone flower” like surface structure could be formed by bonding the L-CNC particles together. The two adhesives had little effect on the surface wettability and oil–water separation properties of the coated meshes. The WCAs were 154.2° (L-CNC/PVDF) and 152.6° (L-CNC/PDMS), respectively, and both the separation efficiency for oil–water separation above 94.5%, meanwhile showing good heat, UV, and acid and alkaline resistance properties. However, they are seriously related to the sandpaper abrasion property. This is because PDMS after curing showed a better flexibility than PVDF after curing and could buffer parts of the external impact force; therefore, PDMS was more beneficial to the abrasion performance. In view of their good oil–water separation ability and mechanical strength as well as facile preparation, the L-CNC coated meshes would have a good application prospect.

## Conflicts of interest

There are no conflicts to declare.

## Supplementary Material
